# Analysis of risk evaluation and mitigation strategies for teratogenic drugs: Variation in primary and secondary prevention measures

**DOI:** 10.1371/journal.pmed.1004190

**Published:** 2023-03-06

**Authors:** Beatrice L. Brown, Aaron S. Kesselheim, Ameet Sarpatwari

**Affiliations:** Program On Regulation, Therapeutics, And Law (PORTAL), Division of Pharmacoepidemiology and Pharmacoeconomics, Department of Medicine, Brigham and Women’s Hospital and Harvard Medical School, Boston, Massachusetts, United States of America

## Abstract

In an analysis of risk evaluation and mitigation strategies for teratogenic drugs, Ameet Sarpatwari, Beatrice Brown and Aaron Kesselheim explore the variation in primary and secondary prevention measures.

**Key points**

- Teratogenicity is a common reason for the US Food and Drug Administration (FDA) to apply risk evaluation and mitigation strategies (REMS) to approved prescription drugs.
- Among the 11 REMS for 10 drugs we analyzed, we found considerable variation in which contraceptives were required, for whom, and when, as well as in the timing and frequency of mandated pregnancy testing, which was only partly explained by the drugs’ known teratogenic risks.
- Further study of the adequacy of REMS for drugs with teratogenic risks may be necessary to optimize their public health benefits.

Since 2007, the US Food and Drug Administration (FDA) has required drug manufacturers to institute risk evaluation and mitigation strategies (REMS) for certain drugs with known safety problems to help ensure that the benefits of use outweigh the risks [1,2]. These REMS may include medication guides for patients, communication plans for prescribers, and more complex elements to assure safe use, such as dispensing restrictions, patient registries, and laboratory testing [3].

Among the most common reasons for a REMS is teratogenicity. In such circumstances, the REMS may include requirements for patients to use contraception (primary prevention) or undergo pregnancy testing (secondary prevention) [4]. Studies examining REMS for drugs with teratogenic risks have reported disparate outcomes [5–8], which may stem from variation in these primary and secondary prevention requirements. Because little scholarship exists on this topic, we sought to compare the requirements of REMS intended to prevent teratogenic risks and their potential effect on patients. The importance of this analysis has increased with the Supreme Court’s decision to overrule *Roe v*. *Wade* in June 2022, [9] and with subsequent enactment of state laws that prohibit abortion even in cases of severe fetal anomalies [10].

For our investigation, we first used the FDA’s REMS database to identify all drugs subject to REMS for teratogenic risks as of September 1, 2021 [11]. For each drug, we recorded the manufacturer, indicated population, and REMS requirements. We then compared these requirements to the known teratogenic risks of the drug. We reviewed the most recent labeling of each drug available on Drugs@FDA [12] and drug-specific data in the Teratogen Information System (TERIS), a widely used commercial database containing information about the teratogenicity of prescription medications [13–17]. Using both resources, we collected evidence of risks of physical malformations (yes, no), organ system malformations (yes, no), and spontaneous abortions (yes, no). Using TERIS, we recorded the listed magnitude of identified teratogenic risks (none, minimal, small, moderate, high, undetermined) and the quantity and quality of data (none, limited, fair, good, or excellent) on which the assessment was based.

For each REMS, we extracted information about the requirements for use of contraception (primary prevention), pregnancy testing (secondary prevention), and emergency contraception (secondary prevention) (Table 1). For use of contraception, we determined the population subject to the requirement, the number and type of methods required, starting and stopping points, and exceptions. For pregnancy testing, we determined the type of pregnancy test required (laboratory-based, unspecified), frequency, starting and stopping points, and exceptions. Finally, for emergency contraception, we determined whether the REMS required the discussion of emergency contraception as part of patient counseling (yes, no) and whether a brochure with this information was required to be provided to the patient (yes, no). We extracted all information as available on September 1, 2021.

**Table 1:** Preventive measures in pregnancy-related risk evaluation and mitigation strategies.

	Adempas	Ambrisentan*	Bosentan*	Isotretinoin*	Lenalidomide*	Macitentan*	*Mycophenolate* *	Pomalidomide*	Pomalyst	*Qsymia*	Thalomid
**Primary prevention**											
Contraceptive requirements for males (in addition to females who can become pregnant)	No	No	No	Yes	Yes	No	No	Yes	Yes	No	Yes
Agreeing to abstinence is not a replacement for the contraceptive requirement	Yes	Yes	Yes	No	No	Yes	No	No	No	Yes	No
Contraceptive requirements before treatment for females	No	No	No	Yes	Yes	No	No	Yes	Yes	No	Yes
Contraceptive requirements after treatment for females	Yes	Yes	Yes	Yes	Yes	Yes	Yes	Yes	Yes	No	Yes
Greater than 1 form of contraception required for all contraceptive methods	No	No	No	Yes	Yes	No	No	Yes	Yes	No	Yes
Hormonal method of contraception required unless tubal ligation or partner’s vasectomy (i.e., no option to use 2 barrier methods)	No	No	No	Yes	Yes	No	No	Yes	Yes	No	Yes
**Secondary prevention**											
Laboratory pregnancy test required	Yes	Yes	Yes	Yes	Yes	Yes	Yes	Yes	Yes	No	Yes
Monthly pregnancy tests required	Yes	Yes	Yes	Yes	Yes	Yes	Yes	Yes	Yes	Yes	Yes
More than 1 pregnancy test required before treatment	No	No	No	Yes	Yes	No	No	Yes	Yes	No	Yes
More frequent than monthly pregnancy testing required at any point following initiation	No	No	No	No	Yes	No	No	Yes	Yes	No	Yes
Agreeing to abstinence is not a replacement for the pregnancy testing requirement	Yes	Yes	Yes	Yes	Yes	Yes	Yes	Yes	Yes	Yes	Yes
Pregnancy test required following treatment for females	Yes	Yes	Yes	No	No	Yes	No	No	No	No	No
Information on emergency contraception must be included by the provider in counseling discussion	No	Yes	No	Yes	Yes	Yes	Yes	Yes	Yes	No	Yes
Information on emergency contraception provided through brochure to patient	No	No	No	No	Yes	No	No	Yes	Yes	No	Yes

Italicized drugs indicate those whose REMS are only recommendations, not requirements.

* Denotes shared REMSs.

Of 62 REMS, we identified 11 (18%) intended to minimize teratogenic risks. Seven applied to the generic and branded versions of drugs to prevent organ transplant rejection (mycophenolate) and to treat pulmonary hypertension (ambrisentan, bosentan, and macitentan), acne (isotretinoin), and multiple myeloma (lenalidomide and pomalidomide). The other 4 REMS applied only to the brand-name product Adempas (riociguat, for pulmonary hypertension), Pomalyst (pomalidomide, for multiple myeloma), Thalomid (thalidomide, for multiple myeloma), and Qsymia (phentermine-topiramate, for weight management). Pomalyst and generic pomalidomide each had their own REMS, which were very similar. The REMS for bosentan managed hepatotoxicity in addition to birth defects, and the REMS for mycophenolate and Qsymia had recommendations instead of requirements.

## Teratogenic risk

The nature of the teratogenic risk varied for the 10 drugs covered by the REMS (Table 2). Six drugs (isotretinoin, lenalidomide, mycophenolate, phentermine-topiramate, pomalidomide, and thalidomide) had evidence of risks of physical malformations, organ system malformations, and spontaneous abortions. Two drugs (ambrisentan and macitentan) had evidence of risks of physical and organ system malformations, but not of spontaneous abortions. One drug (riociguat) was linked to organ system malformations and spontaneous abortions but did not have evidence of risk of physical malformations, and 1 drug (bosentan) had evidence of risk of physical malformations alone.

**Table 2:** Data on teratogenicity.

Drug name	Data source		Evidence of			Magnitude of teratogenic risk to child born after exposure during gestation	Quantity and quality of data on which risk estimate is based	Comment from TERIS database
	Animals	Humans	Physical malformations	Organ system malformations	Spontaneous abortion			
Ambrisentan	Yes	No	Yes	Yes	No	Undetermined	None	N/A
Bosentan	Yes	No	Yes	No	No	Undetermined	Very limited	N/A
Isotretinoin	Yes	Yes	Yes	Yes	Yes	High	Excellent	“This rating is for systemic (oral) treatment with isotretinoin.”
Lenalidomide*	Yes	No	Yes	Yes	Yes	Undetermined	Limited	“Although the risk of lenalidomide is undetermined, it is likely to be substantial because lenalidomide is an analog of thalidomide and has antineoplastic activity.”
Macitentan	Yes	No	Yes	Yes	No	Undetermined	None	“Although undetermined, the fetal risk associated with material use of this drug during early pregnancy may be substantial because macitentan is an endothelin-1 receptor antagonist and this pathway is important in normal embryogenesis.”
Mycophenolate	Yes	Yes	Yes	Yes	Yes	Moderate-to-high	Fair-to-good	N/A
Phentermine-Topiramate	Yes	No	No	No	No	Undetermined	Limited	“The risk of congenital anomalies may be increased in the children of obese women.”
	Yes	Yes	Yes	Yes	Yes	Small	Fair-to-good	N/A
Pomalidomide*	Yes	No	Yes	Yes	Yes	Undetermined	Very limited	“Although the risk of pomalidomide is undetermined, it is likely to be substantial because pomalidomide is an analog of thalidomide and has antineoplastic activity.”
Riociguat	Yes	No	No	Yes	Yes	Undetermined	None	“Although undetermined, the fetal risk associated with maternal riociguat treatment during early pregnancy may be substantial because this drug stimulates soluble guanylate cyclase, which is important in normal cardiac development in the embryo. In addition, unpublished studies performed by the manufacturer are said to have shown increased rates of ventricular septal defects among the offspring of pregnant rats treated with riociguat in doses that produced blood levels within the human therapeutic range.”
Thalidomide	Yes	Yes	Yes	Yes	Yes	High	Excellent	N/A

* Thalidomide analogue.

For 4 drugs (isotretinoin, mycophenolate, phentermine-topiramate, and thalidomide), human data supported the teratogenic risks. TERIS reported that 2 drugs (isotretinoin and thalidomide) had a high magnitude of teratogenic risk and excellent quantity and quality of evidence. Mycophenolate had a moderate-to-high magnitude of risk and fair-to-good quantity and quality of evidence, while topiramate—one component of phentermine-topiramate—had a small magnitude of risk and fair-to-good quantity and quality of evidence. The remaining 6 drugs had an undetermined magnitude of risk, with quantity and quality of data ranging from none (ambrisentan, macitentan, riociguat), to very limited (bosentan, pomalidomide), to limited (lenalidomide). For 3 of these drugs, TERIS noted the potential for high teratogenicity because of their drug class (lenalidomide, pomalidomide) or mechanism of action (riociguat).

Thus, most drugs with REMS for teratogenicity lacked human data on teratogenicity. Of these drugs, most also had low TERIS ratings for quantity and quality of data and an undetermined magnitude of risk.

### Primary prevention

Five REMS (for isotretinoin, lenalidomide, pomalidomide, Pomalyst, and Thalomid) required the use of contraception by males and females, and 5 REMS (for Adempas, ambrisentan, bosentan, macitentan, and Qsymia) made no exceptions for abstinence from sexual activity. Five REMS (isotretinoin, lenalidomide, pomalidomide, Pomalyst, and Thalomid) required contraception to be used at least 4 weeks before initiating treatment, and 10 REMS (all except for Qsymia) required or recommended contraception use following drug discontinuation.

Five REMS (for isotretinoin, lenalidomide, pomalidomide, Pomalyst, and Thalomid) required multiple methods of contraception, one of which was hormonal, unless the female had undergone tubal ligation (or her male partner a vasectomy); neither intrauterine devices (IUDs) nor tubal sterilization alone were sufficient. Two REMS (for bosentan and mycophenolate) did not require or recommend an additional method of contraception with an IUD but did with a progesterone implant, while 5 REMS (for isotretinoin, lenalidomide, pomalidomide, Pomalyst, and Thalomid) listed unacceptable contraception methods, including progesterone-only mini-pills, the Progesterone T hormonal IUD, and female condoms.

To promote compliance with these primary prevention requirements, 9 REMS (all except for mycophenolate and Qsymia) required patients and prescribers to sign attestations of understanding. However, only the REMS for isotretinoin required that prescribers and patients document the 2 methods of contraception being used.

Overall, the REMS for isotretinoin, lenalidomide, pomalidomide, Pomalyst, and Thalomid had the most stringent primary prevention requirements. They mandated contraceptive use before treatment initiation and after treatment discontinuation, use of more than 1 form of contraception for all contraceptive methods, and use of hormonal methods of contraception absent tubal ligation.

### Secondary prevention

Every REMS required or recommended at least monthly pregnancy testing of all females of reproductive potential, without exceptions for abstinence, and every REMS except for Qsymia required or recommended lab-based pregnancy testing. Nine REMS (all except for mycophenolate and Qsymia) ensured compliance with the pregnancy testing requirement by making dispensing dependent upon a check by the prescriber and/or pharmacist for a negative test result. Five REMS (for isotretinoin, lenalidomide, pomalidomide, Pomalyst, and Thalomid) required multiple tests at various intervals before treatment initiation; 4 REMS (for lenalidomide, pomalidomide, Pomalyst, and Thalomid) required weekly testing during the first month of treatment, and 4 REMS (for Adempas, ambrisentan, bosentan, and macitentan) required at least 1 pregnancy test after discontinuation of treatment.

Seven REMS (for ambrisentan, isotretinoin, lenalidomide, macitentan, pomalidomide, Pomalyst, and Thalomid) required prescribers to discuss emergency contraception during counseling (this was only a recommendation for mycophenolate). Of these REMS, 4 (for lenalidomide, pomalidomide, Pomalyst, and Thalomid) further required the prescriber to provide the patient with literature about emergency contraception.

Thus, as with the primary prevention requirements (with the exception of the REMS for isotretinoin)., the REMS for lenalidomide, pomalidomide, Pomalyst, and Thalomid had the most stringent secondary prevention requirements. These included more than 1 pregnancy test before treatment initiation and dissemination of a brochure on emergency contraception during counseling.

### REMS stringency may not align with teratogenic risk

Our analysis of 11 REMS for 10 teratogenic drugs reveals substantial variation as to which contraceptives were required, for whom, and when, as well as to the timing and frequency of mandated pregnancy testing. Some of this variation may be due to differences in the drugs and their activity. The REMS for isotretinoin, lenalidomide, pomalidomide, Pomalyst, and Thalomid, for example, required that males taking the drug agree to condom use during sex. Preclinical testing of these drugs had revealed possible drug transmission via blood or semen.

The statutory factors that the FDA must consider when deciding to implement a REMS—“(1) the estimated size of the population likely to use the drug; (2) the seriousness of the indicated disease or condition; (3) the expected benefit of the drug; (4) the expected or actual duration of treatment; (5) the seriousness of any known or potential adverse events that may be related to the drug and the background incidence of such events in the target population; and (6) whether the drug is a new molecular entity” [18]—may also explain some variation in REMS requirements. However, even when accounting for these factors, reasons for the stringency of the REMS requirements for some drugs were unclear. For example, although there was evidence of mycophenolate causing physical malformations, organ system malformations, and spontaneous abortion in humans, its REMS comprised recommendations rather than requirements. Similarly, animal data for mycophenolate revealed possible drug transmission via blood or semen, but the REMS was not updated to require male condom use. This misalignment of REMS requirements with teratogenic risks may be due to the fact that the patient population taking the drug for its approved indication—a prophylaxis for organ transplant rejection—is small. However, many patients take mycophenolate for unapproved indications, like lupus, in accordance with professional treatment guidelines [19]. By contrast, lenalidomide, pomalidomide, and thalidomide had restrictive REMS, but few females of reproductive potential have multiple myeloma.

Other REMS may not have gone far enough to prevent pregnancy. The REMS for Adempas, ambrisentan, bosentan, macitentan, mycophenolate, and Qsymia did not require that females begin contraceptives before treatment initiation. However, some methods of contraception permitted under these REMS, including combined oral contraceptives, may leave a gap between treatment initiation and full protection against pregnancy since they are not effective immediately. Additionally, the REMS for Adempas, bosentan, and Qsymia did not include counseling females on emergency contraception, which can help prevent fetal-embryo exposure in the case of contraceptive failures.

### REMS requirements and patient autonomy

It is important to consider how REMS requirements impact patient autonomy. For example, the REMS for isotretinoin, lenalidomide, pomalidomide, Pomalyst, and Thalomid required 2 methods of contraception for females regardless of which primary method was used, including an IUD. However, in routine clinical settings, females are counseled that a second method of birth control is not necessary for avoiding pregnancy following the insertion of an IUD or hormonal implant [20].

Additionally, several REMS did not permit the use of 2 barrier methods of contraception and instead required at least 1 hormonal method or another invasive method, such as a nonhormonal IUD, tubal ligation, or partner’s vasectomy. Hormonal methods of contraception can have side effects, ranging from nausea, headache, irritability, and weight gain to more serious thromboembolic events [21]. Certain contraception methods, such as combined oral contraceptive pills, are contraindicated for some patients due to an elevated risk of stroke or other cardiovascular adverse events, such as those who suffer from migraine with aura, or are strongly recommended against for those with certain medical conditions, such as a history of hypertension [22]. These populations can usually use other methods that do not contain estrogen [21], but such methods may either be more invasive or not permitted under the REMS (such as progestin-only pills). For example, IUDs require an insertion process that patients may not want to undergo and that can result in rare but severe complications, such as device expulsion and uterine perforation [23]. Tubal ligation is irreversible, and barriers to access, including discouragement by clinicians, [24] may deter women who wish to undergo the procedure.

### Opportunities to improve REMS for teratogenic risks

REMS intended to minimize teratogenic risks must optimally balance risk minimization with patient autonomy. The FDA might consider allowing females of reproductive potential taking these drugs to comply with the contraception requirement without needing to use either hormonal or invasive nonhormonal methods. For example, such REMS could allow females to use 2 barrier nonhormonal methods or to agree to abstinence to ensure that females can access the drug in question even if they cannot use hormonal methods or are unwilling to for health or religious reasons. Some REMS, such as those for Adempas, ambrisentan, and bosentan, already allow for 2 barrier nonhormonal methods to be used, and others, including isotretinoin, lenalidomide, and Pomalyst, allow abstinence in lieu of other contraceptive methods. Any changes to REMS related to teratogenic risks should be followed up to ensure they do not increase pregnancies.

For secondary prevention, the heightened frequency of testing before treatment initiation and during the first month of treatment for some REMS may pose cost and access issues for patients without meaningfully contributing to risk mitigation [25]. In addition to streamlining these testing requirements, REMS should ensure that females are effectively counseled on emergency contraception and provided with take-home materials to foster patient autonomy in minimizing teratogenic risks.

There is substantial variation in primary and secondary prevention measures across REMS that intend to minimize teratogenic risks. Some of this variation may be explained by differences in the drug or statutory factors that the FDA is required to consider. To protect reproductive autonomy in an increasingly fraught political climate, the FDA can reexamine REMS for drugs with teratogenic risks to ensure their requirements are aligned with available evidence on safety.
